# Imaging characteristics of central neurocytomas according to Ki-67 proliferation index

**DOI:** 10.3389/fonc.2025.1601817

**Published:** 2025-06-03

**Authors:** Yan-li Zhang, Ling Liu, Wei Li, Chao Ran, Yu-qi Luo

**Affiliations:** ^1^ Department of Clinical Pharmacy, Affiliated Hospital of Yangzhou University, Yangzhou University, Yangzhou, China; ^2^ Obstetrics Department, Affiliated Yantai Yuhuangding Hospital of Qingdao University, Yantai, China; ^3^ Medical Imaging Department, Affiliated Hospital of Yangzhou University, Yangzhou University, Yangzhou, China; ^4^ Department of Radiology, Affiliated Yantai Yuhuangding Hospital of Qingdao University, Yantai, China; ^5^ Department of Radiology, Beijing Tiantan Hospital, Capital Medical University, Beijing, China

**Keywords:** central neurocytoma, Ki-67, MRI, CT, imaging diagnosis

## Abstract

**Purpose:**

To evaluate the imaging characteristics of central neurocytoma (CN) with different Ki-67 indices and reveal its biological behavior.

**Materials and methods:**

Sixty-nine cases of intraventricular CN confirmed by histopathology were collected retrospectively. According to Ki-67 indices, they were divided into CNs with high Ki-67 (30 cases) and CNs with low Ki-67 (39 cases). Their clinical and imaging findings were compared and analyzed.

**Results:**

Compared to CNs with low Ki-67, CNs with high Ki-67 were larger (P < 0.001) and more solid (P = 0.019). Restricted diffusion (P = 0.020), CT hyperdensity (P < 0.001), abnormal vessels (P = 0.018), and marked MRI enhancement (P < 0.001) were more common in CNs with high Ki-67.

**Conclusion:**

Different Ki-67 indices could affect the imaging characteristics of CNs. Tumor size, radiologic solidity, abnormal vessels, and marked MRI enhancement suggested CNs with high Ki-67. Combining these imaging characteristics and Ki-67 indices offered meaningful insights into their clinical diagnosis and biological behavior.

## 1 Introduction

Central neurocytoma (CN) is a rare neuronal and mixed neuronal-glial tumor (WHO grade II), accounting for approximately 0.1% - 0.5% of primary brain tumors (1, 2). It is classically located in the lateral ventricle around the foramen of Monro in young adults aged 20–40 years (3, 4). Most of the clinical symptoms are caused by obstructive hydrocephalus and intracranial hypertension, including dizziness, headache, nausea, vomiting, papilledema, and ataxia (5).

Ki-67 index is a reliable marker for cell growth and tissue differentiation (6). Tumors with a low Ki-67 index exhibit good biological behavior, while an elevated Ki-67 index suggests malignant proliferation (6, 7). Most reports indicated that CNs had limited invasiveness, and total resection is an effective treatment with a good prognosis (3, 4, 8). However, the high Ki-67 index may induce atypical histological features and malignant potential (1, 9, 10). These conditions complicate the understanding of CN’s biological behavior, and postoperative radio chemotherapy is suggested (9–11). Accurate preoperative diagnosis is important for clinical intervention. Although atypical CNs can manifest as heterogeneous extra ventricular lesions with parenchymal invasion and cerebral edema, the relationship between Ki-67 index and intraventricular CN has not been systematically evaluated. We hypothesize that different Ki-67 indices may affect imaging characteristics of intraventricular CNs. Therefore, this study aims to provide new insights into their preoperative diagnosis and biological behavior by comparing CNs with different Ki-67 indices.

## 2 Materials and methods

### 2.1 Patients and imaging examinations

Eighty-one patients with CN confirmed by pathology from June 2014 to July 2024 were collected. The pathological diagnosis was reached by histomorphological and immunohistochemical features. Inclusion criteria: definite pathological and immunohistochemical diagnoses, definite Ki-67 index, and complete clinical and imaging data. Exclusion criteria: concomitant with other brain tumors, preoperative chemoradiotherapy, poor imaging quality, postoperative recurrence, and extraventricular neurocytoma. All patients underwent magnetic resonance imaging (MRI) using 3.0 T MRI units (GE Signa or GE Discovery, Milwaukee, WI, USA, slice thickness = 5mm, slice interval = 1.5 mm, matrix = 256 × 256 or 512 × 512), including T1-weighted imaging (T1WI, TR = 1750 ms, TI = 720–760 ms), T2-weighted imaging (T2WI, TR = 4000–5000 ms, TE = 100–120 ms), fluid-attenuated inversion recovery (FLAIR, TR = 8000–8400 ms, TI = 2000–2100 ms, TE = 120 ms), and diffusion-weighted imaging (DWI, b values = 0 and 1000s/mm^2^, TR = 3000–500 ms, TE = 70–100 ms). After intravenous gadolinium injection (0.2 ml/kg), the contrast-enhanced T1WI was obtained. These patients also underwent unenhanced computed tomography (CT, 16 - or 64 - section; GE Hispeed, Milwaukee, WI, USA, tube voltage = 120 kV, tube current = 100–400 mA, slice thickness = 5 mm, slice interval = 5 mm) before treatment. CT imaging is a fast and low-cost preliminary screening before MRI. Especially, some patients came for treatment with intracranial hypertension, and CT was the preferred emergency imaging method. On time-saving and low-cost grounds, X-ray exposure was deemed ethically and clinically acceptable in this study. According to the inclusion and exclusion criteria, 12 cases were ultimately excluded, including 4 cases of incomplete clinical and imaging data, 3 cases of extraventricular neurocytoma, 3 cases of preoperative chemoradiotherapy, and 2 cases of postoperative recurrence. The remaining 69 cases underwent clinical and imaging analyses.

### 2.2 Clinical and imaging analyses

Based on the Ki-67 indices, 69 patients were divided into CN with high Ki-67 (Ki-67 index > 3%) and CN with low Ki-67 (Ki-67 index ≤ 3%) (12, 13). Their clinical and imaging data were collected and compared. The clinical and imaging indicators included age, sex, intracranial hypertension (dizziness, headache, nausea, or vomiting), size, apparent diffusion coefficient (ADC) value, CT density, cystic-solidity (solid or multi-cystic), intratumoral hemorrhage, intratumoral calcification, marked MRI enhancement, hydrocephalus, periventricular edema, and abnormal vessels. Hyperintensity on DWI and hypointensity on the ADC map suggested restricted diffusion. Avoiding intratumoral calcification, hemorrhage, cystic changes, and abnormal vessels, the CT density and ADC value were measured at the solid part of the lesion. The region of interest (ROI) was determined by the cross-sectional shape of the lesion. ROIs of 20–100 mm^2^ were suitable for all cases in this study. Furthermore, the mean value of the three ROIs at different positions was selected to ensure the reliability of the measurement. The enhancement degree of the lesion equal to or greater than that of the choroid plexus was defined as marked MRI enhancement. All imaging analyses were performed independently by two neuroradiologists with ten years of experience without knowing the pathological results. In case of different opinions, a consensus should be reached through negotiation.

### 2.3 Statistical analysis

Statistical analyses were performed to characterize the clinical and imaging features by IBM SPSS 26.0 (SPSS, Chicago, IL, USA). All data were presented as numbers (percentage) or mean ± SD. According to the different data distribution, an independent t-test (two-tailed) or Mann-Whitney U-test was used for the continuous variables, and a chi-square test or Fisher’s exact test (two-tailed) was used for dichotomous variables. P < 0.05 was defined as statistically significant. The interclass correlation coefficient (ICC) or Kappa value was used for the inter-observer variability assessment. ICC > 0.75 or Kappa ≥ 0.8 suggested a better agreement.

## 3 Results

**Figure 1:**
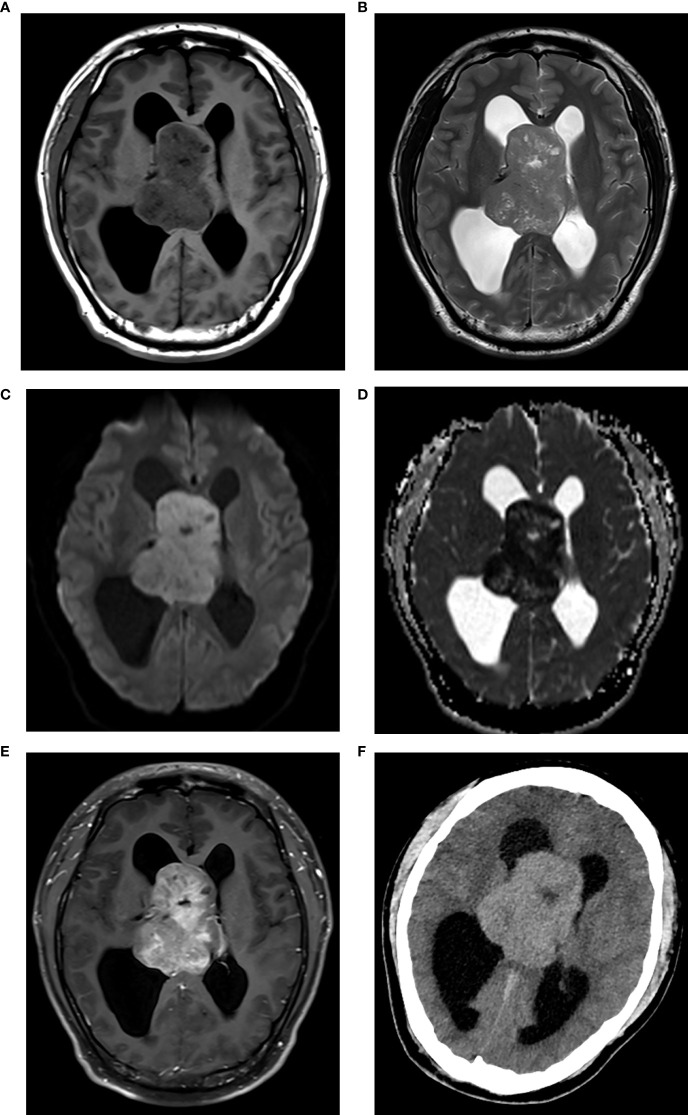
CN with high Ki-67. Axial MRI scans revealed a solid lesion around the septum pellucida, showing isointensity on T1WI **(A)** isointensity on T2WI **(B)** hyperintensity on DWI **(C)** hypointensity on ADC map [**(D)** the mean ADC = 0.73×10–^3^ mm^2^/s], and marked enhancement on contrast-enhanced T1WI **(E)**. The lesion protruded into both lateral ventricles and caused hydrocephalus. The lesion showed hyperdensity on axial CT [**(F)**, CT density = 46 Hu].

**Figure 2:**
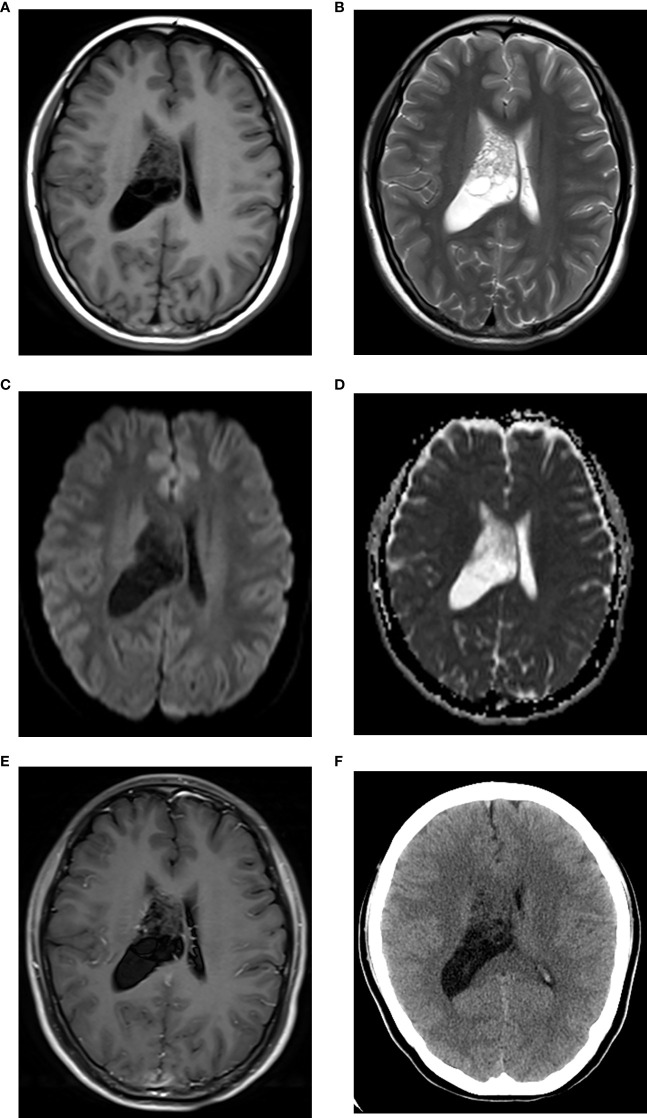
CN with low Ki-67. Axial MRI scans revealed a multi-cystic lesion in the right lateral ventricle, showing iso-hypointensity on T1WI **(A)** iso-hyperintensity on T2WI **(B)** iso-hypointensity on DWI **(C)** iso-hyperintensity on ADC map [**(D)** the mean ADC = 1.59×10–^3^ mm^2^/s], and without marked enhancement on contrast-enhanced T1WI **(E)**. The right lateral ventricle was enlarged. The lesion showed iso-hypodensity on axial CT [**(F)**, CT density = 28 Hu].

The Ki-67 index of all 69 cases did not exceed 10%, 39 cases (56.5%) were no more than 3%, and the others (30 cases, 43.5%) were 4% to 10%. The inter-observer agreement was good in clinical and imaging analyses (ICC = 0.87 or Kappa = 0.90). In this study, all CN lesions were located in the lateral ventricle or extended to the contralateral ventricle or the third ventricle, causing hydrocephalus and intracranial hypertension. Compared with CNs with low Ki-67, CNs with high Ki-67 were larger (60.33 ± 35.17 cm^3^ *vs* 15.56 ± 9.79 cm^3^, P < 0.001) and more solid (53.3% *vs* 25.6%, P = 0.019). Restricted diffusion (0.85 ± 0.14 ×10–^3^ mm^2^/s *vs* 1.48 ± 0.22 ×10–^3^ mm^2^/s, P = 0.020), CT hyperdensity (43.20 ± 5.71 Hu *vs* 34.46 ± 7.49 Hu, P < 0.001), abnormal vessels (33.3% *vs* 10.3%, P = 0.018) and marked MRI enhancement (66.7% *vs* 17.9%%, P < 0.001) were more common in CNs with high Ki-67. There were no differences in age, sex, intracranial hypertension, hydrocephalus, intratumoral hemorrhage, intratumoral calcification, and periventricular edema between the two groups. See **Table 1**, **Figures 1**, **2** for details.

**Table 1:** Clinical and imaging comparisons between the two groups.

Clinical and imaging indicators	CN with high Ki-67 (n = 30)	CN with low Ki-67 (n = 39)	P value
Age (years)	33.8 ± 3.9	34.4 ± 6.2	0.085
Sex (Male/Female)	17/13	18/21	0.387
Intracranial hypertension	23 (76.7%)	26 (66.7%)	0.364
Size (cm^3^)	60.33 ± 35.17	15.56 ± 9.79	< 0.001^*^
ADC value (×10–^3^ mm^2^/s)	0.85 ± 0.14	1.48 ± 0.22	0.020^*^
CT density (Hu)	43.20 ± 5.71	34.46 ± 7.49	< 0.001^*^
Intratumoral hemorrhage	4 (13.3%)	2 (5.1%)	0.442
Cystic-solidity (solid/multi-cystic)	16/14	10/29	0.019^*^
Intratumoral calcification	15 (50%)	19 (49.3%)	0.916
Marked MRI enhancement	20 (66.7%)	7 (17.9%)	< 0.001^*^
Hydrocephalus	22 (73.3%)	23 (59.0%)	0.214
Periventricular edema	2 (6.7%)	0	0.185
Abnormal vessels	10 (33.3%)	4 (10.3%)	0.018^*^

CN, central neurocytoma; ADC, apparent diffusion coefficient; CT, computed tomography; MRI, magnetic resonance imaging. Values are given as n, n (%), or mean ± SD. *Significance values.

## 4 Discussion

CN originates from bipotential progenitor cells with neuronal and glial differentiation (14, 15). The Ki-67 index is no more than 3% in normal cells and more than 10% in malignant tumors (16, 17). The cut-off value of the Ki-67 index for the atypical CN ranges from 2% to 5% (18, 19). The Ki-67 index > 3% is often used as a threshold for the pathological grading of CN (1, 12, 13). In this study, the Ki-67 indices were 4% to 10% in CNs with high Ki-67. Our results further demonstrated that different Ki-67 indices could affect the imaging characteristics of CN and reveal its biological behavior.

CNs with high Ki-67 indices were often large solid lesions, implying a high tumor burden. The hypercellularity caused by high Ki-67 indices determined the radiologic solidity of CN (20). Limited extracellular space, hypercellularity, and high nucleus-cytoplasm ratio resulted in CT hyperdensity and restricted diffusion (21, 22). The abundant blood supply from the choroidal arteries might also prevent necrosis and cystic changes. However, CNs with low Ki-67 indices were mostly multi-cystic lesions, suggesting a tendency for gangliocyte differentiation (23). The central cystic changes were often caused by tumor necrosis, while the peritumoral cystic changes were due to adhesion, traction, and cerebrospinal fluid infiltration (24). Elevated Ki-67 index also represented active vascularization, bringing about abnormal blood vessels of CN (25). Of course, these abnormal vessels might come from the encapsulation of adjacent blood vessels during CN growth. Susceptibility-weighted imaging (SWI) or T2*WI is sensitive to the vascular components and intratumoral susceptibility signals of CNs with high Ki-67, contributing to a more accurate diagnosis (26–28). The marked enhancement could be explained by hypercellularity and tumor angiogenesis, which were also related to elevated Ki-67 indices (29, 30). The solid lesions with CT hyperdensity and restricted diffusion represented the hypercellularity. Abnormal vessels and marked MRI enhancement meant hypervascularity. Therefore, combining the high Ki-67 index, hypercellularity, and hypervascularity could further reveal the complex biological behavior of CN.

In this study, these patients were young and middle-aged without significant sex differences. Age is a possible influencing factor for the histological grading of CN. Extreme age (>50 years or < 18 years) might tend to atypical features and recurrence (31). The age of our patients did not show this extreme tendency, which needs to be further confirmed by a large sample. The clinical symptoms were associated with the location and mass effect of the lesion (5, 32). Both groups of CN caused obstructive hydrocephalus and intracranial hypertension, although CNs with high Ki-67 were larger. All the lesions near the foramen of Monro were the possible reason. Moreover, the slow growth of CN could compensate for intracranial hypertension and reduce periventricular edema. Intratumoral calcification and hemorrhage were not associated with the Ki-67 indices in this study. Although the blood supply of CN was abundant, intratumoral hemorrhage was uncommon (33). The unbalanced blood supply and vascular degeneration might result in intratumoral hemorrhage and calcification (33, 34).

The imaging assessment of CN based on the Ki-67 index provided clinical implications. The combination of Ki-67 index and imaging characteristics was helpful for preoperative diagnosis and postoperative follow-up of CN. The atypia and hypercellularity related to the high Ki-67 index can lead to postoperative recurrence and poor prognosis (9, 11, 14). Total resection and postoperative radiochemotherapy are recommended. However, the hypervascular CN with high Ki-67 index also increases the difficulty of total resection, and emergency preparation for intraoperative bleeding is necessary (9, 11, 14). In addition, SWI and T2*WI should be used in preoperative diagnosis and postoperative follow-up to better display abnormal blood vessels.

Due to the low incidence of CN, the small sample size limited the statistical power. Indeed, a single Ki-67 index can not fully reflect the complex biological behavior of CN. SWI and T2*WI were not routine sequences in our institution, so they were not included in this retrospective study. In the future, large-scale longitudinal studies with more tumor markers and advanced imaging techniques are necessary.

## 5 Conclusion

According to the Ki-67 proliferation index, the imaging characteristics of CNs could be affected. Tumor size, radiologic solidity, abnormal vessels, and marked MRI enhancement helped distinguish CNs with different Ki-67 indices. Combining these imaging characteristics with the Ki-67 index offered meaningful insights for clinical diagnosis and biological behavior of CNs.

## Data Availability

The original contributions presented in the study are included in the article/supplementary material. Further inquiries can be directed to the corresponding author.
